# Introduction of an electronic monitoring system for monitoring compliance with Moments 1 and 4 of the WHO "My 5 Moments for Hand Hygiene" methodology

**DOI:** 10.1186/1471-2334-11-151

**Published:** 2011-05-26

**Authors:** Vincent CC Cheng, Josepha WM Tai, Sara KY Ho, Jasper FW Chan, Kwan Ngai Hung, Pak Leung Ho, Kwok Yung Yuen

**Affiliations:** 1 Department of Microbiology, Queen Mary Hospital, Hong Kong Special Administrative Region, China; 2 Infection Control Team, Queen Mary Hospital, Hong Kong Special Administrative Region, China; 3 Department of Neurosurgery, Queen Mary Hospital, Hong Kong Special Administrative Region, China

## Abstract

**Background:**

MedSense is an electronic hand hygiene compliance monitoring system that provides Infection Control Practitioners with continuous access to hand hygiene compliance information by monitoring Moments 1 and 4 of the WHO "My 5 Moments for Hand Hygiene" guidelines. Unlike previous electronic monitoring systems, MedSense operates in open cubicles with multiple beds and does not disrupt existing workflows.

**Methods:**

This study was conducted in a 6-bed neurosurgical intensive care unit with technical development and evaluation phases. Healthcare workers (HCWs) wore an electronic device in the style of an identity badge to detect hand hygiene opportunities and compliance. We compared the compliance determined by the system and an infection control nurse. At the same time, the system assessed compliance by time of day, day of week, work shift, professional category of HCWs, and individual subject, while the workload of HCWs was monitored by measuring the amount of time they spent in patient zones.

**Results:**

During the three-month evaluation phase, the system identified 13,694 hand hygiene opportunities from 17 nurses, 3 physiotherapists, and 1 healthcare assistant, resulting in an overall compliance of 35.1% for the unit. The per-indication compliance for Moment 1, 4, and simultaneous 1 and 4 were 21.3% (95%CI: 19.0, 23.6), 39.6% (95%CI: 37.3, 41.9), and 49.2% (95%CI: 46.6, 51.8), respectively, and were all statistically significantly different (p < 0.001). In the four 20-minute sessions when hand hygiene was monitored concurrently by the system and infection control nurse, the compliance were 88.9% and 95.6% respectively (p = 0.34), and the activity indices were 11.1 and 12.9 opportunities per hour, respectively. The hours from 12:00 to 14:00 had a notably lower compliance (21.3%, 95%CI: 17.2, 25.3) than nearly three quarters of the other periods of the day (p < 0.001). Nurses who used shared badges had significantly (p < 0.01) lower compliance (23.7%, 95%CI: 17.8, 29.6) than both the registered nurses (36.1%, 95%CI: 34.2, 37.9) and nursing officers (34.0%, 95%CI: 31.1, 36.9) who used named badges.

**Conclusion:**

MedSense provides an unobtrusive and objective measurement of hand hygiene compliance. The information is important for staff training by the infection control team and allocation of manpower by hospital administration.

## Background

Healthcare-associated infection (HCAI) poses a great threat to patient safety in a modern healthcare system. At any point in time, up to 8% of patients in acute care hospital have HCAI, while 19% of patients may have the manifestation of infection after hospital discharge as shown in a cohort study of 4000 patients in UK [1]. In the USA, HCAI is amongst the top ten causes of death [2,3]. The US Institute of Medicine estimated that preventable adverse patient events, including HCAIs, were responsible for 44,000-98,000 deaths annually in the US at a cost of $17-$29 billion [4].

For these reasons, the World Health Organization (WHO) in 2005 initiated the 'First Global Patient Safety Challenge' aiming to improve patient safety by promoting hand hygiene in healthcare facilities with a multimodal strategy that included the use of alcohol-based handrub at the point of care [5]. Hand hygiene is always considered the cornerstone practice to reduce HCAIs as most pathogen transmission is mediated by the hands of healthcare workers (HCWs) [6]. Numerous studies have demonstrated the benefit of alcohol-based handrub in terms of reducing incidence of endemic infection such as MRSA and outbreak controls [7-11]. However, the main hurdle remains the poor hand hygiene compliance of our HCWs, especially in clinical settings with high intensity of patient care such as intensive care units. There have been different strategies in enhancing hand hygiene such as our recent introduction of directly observed hand hygiene practice [8,9]. Another commonly adopted strategy has been regular hand hygiene observation with performance feedback, which is rather labor intensive.

Introduction of an electronic device for monitoring hand hygiene practice can be a simple but innovative idea in solving our limitation in manpower. In fact, previous works have described the use of electronic monitoring systems to record entry and exit from patient rooms as well as frequency of alcohol or soap dispenser use [12-18]. However, the detail of patient care practice cannot be captured by these systems. To our understanding, this is the first study to introduce an electronic system to monitor the compliance of hand hygiene based on the WHO recommended guidelines among HCWs in open cubicles with multiple beds.

## Methods

### Electronic monitoring system - MedSense

MedSense (General Sensing Limited, Hong Kong SAR, China) is an innovative electronic compliance monitoring system that was created to help healthcare institutions achieve improvements in hand hygiene performance through continuous and automated monitoring. The system includes a family of electronic devices that work together with analysis software and web-based reporting tools to provide performance feedback to the healthcare institution.

MedSense devices communicate through a proprietary wireless sensor protocol that allows for low power yet high-resolution proximity detection between devices. The underlying 2.4 GHz radio frequency (RF) technology has been tested to comply with Code of Federal Regulations, Title 47, Part 15 related to transmitted power and interference, and previous research conducted in hospitals using the system has not resulted in any observed interference [19].

**Figure 1:**
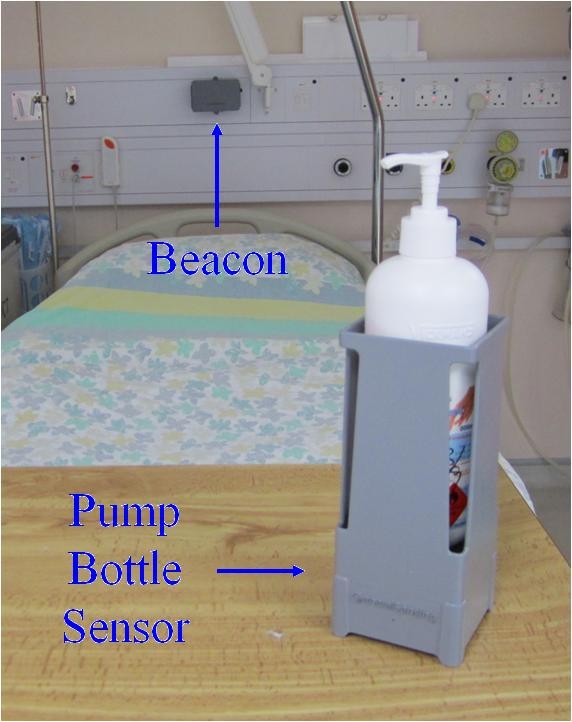
**MedSense devices including beacon and pump bottle sensor at the bedside**.

Unlike other electronic monitoring systems, MedSense was designed to monitor HCWs following the WHO "My 5 Moments for Hand Hygiene" guidelines. Specifically, MedSense addresses two of the five moments: "Before Touching a Patient" (Moment 1) and "After Touching a Patient" (Moment 4). To accomplish this, the system requires that HCWs wear an electronic badge, which is styled after an identity card. The badge detects hand hygiene by sensing proximity to beacons that create patient zones around individual patient beds, and it detects hand hygiene actions by sensing proximity to sensors embedded in soap and alcohol dispensers that are activated when the dispenser is used. Network-connected base stations wirelessly upload the badges' stored sensor data to a server, which processes hand hygiene opportunities and actions into compliance data. Figure 1 shows the MedSense family at the bedside and Table 1 shows the technical details of MedSense's information flow.

**Table 1:** Technical details of MedSense's information flow

*Opportunity Detection*
MedSense detects opportunities for hand hygiene in four steps: (i) badges detect "events" when the HCW moves in and out of patient zone; (ii) events are assigned a probability of patient contact based on duration; (iii) events with high probability of patient contact are split into "Before Touching a Patient" and "After Touching a Patient" hand hygiene indications; and (iv) isolated indications are counted as opportunities while "After Touching a Patient" indications followed by "Before Touching a Patient" indications in quick succession are combined into single opportunities.

*Event Detection*

The system defines an "event" as an interval when a badge-wearing HCW spent time in a patient zone within range of a Beacon installed on the wall at the head of the patient's bed. The Beacons, which focus their transmissions into an elliptical field around the bed, periodically broadcast, and the Badges receive these "pings" and record the patient zone ID and signal strength. The "Received Signal Strength Indication" (RSSI) of the ping functions as an indicator of distance between the two devices. During the technical phase of the trial, the Beacons were calibrated such that a patient zone extending approximately arm's length from the bed's perimeter could be detected by applying a threshold to the RSSI (Figure 6). A detection algorithm inputs these ping data points and calibration values and outputs a series of events defined by start and stop times together with the patient zone and badge ID. The algorithm uses a timeout of one minute where a badge may leave the patient zone and return while continuing the active event.

*Patient Contact Inference*

MedSense uses a predefined reference table to predict the probability of patient contact having occurred during an event. The table is indexed by event duration, and the corresponding probability value represents the probability of patient contact during the event. The table's values derive from the results of data observation on the unit, which showed a strong relationship between the duration spent in a patient zone and patient contact occurring. Figure 7 shows the probability of patient contact in relation to the event duration. Events with a low probability of patient contact (duration less than fifteen seconds) are disregarded, and the remaining events each create two indications for hand hygiene: "Before Touching a Patient" and "After Touching a Patient", which are assigned times equal to the start and end times of the events, respectively. In addition to type and time, the indications carry forward their probability of patient contact as a weighting factor to be used in the compliance calculation.

*Opportunity Algorithm*

According to the WHO's recommendation, the occurrence of a single indication creates an opportunity for hand hygiene. MedSense therefore counts each isolated indication as an opportunity with probability of occurrence equal to the indication probability. When multiple indications occur at the same time, the WHO specifies that only a single opportunity should be counted. MedSense groups an "After Touching a Patient" followed by a "Before Touching a Patient" indication that happen within two minutes of each other as a single opportunity. When combining these two indications, the resulting opportunity has a probability equal to the probability that at least one of the two indications occurred.

*Action Detection*

Wireless sensors detect when HCWs dispense alcohol and soap product, and then broadcast an activation message to proximate badges, which record the messages. The action algorithm selects activation messages with strong signal strength and assigns them to badges as hand hygiene actions.

*Activation Detection*

Pump bottle sensors accommodate a single alcohol or soap bottle. The sensors can be mounted on a wall or placed on a flat surface. When the HCW presses on a bottle's pump to dispense product, a force sensor module in the bottom of the unit triggers and broadcasts a message indicating that a pump bottle "activation" occurred.

*Action Algorithm*

Each badge that receives a particular activation message records the identifying information together with the time and RSSI. When this data is uploaded to the server, the action algorithm selects activations with an RSSI above a threshold as representing badges, and therefore HCWs, who could have initiated the hand hygiene action. When a single activation is selected for a particular action, the action algorithm directly assigns it to the corresponding badge. If multiple activations are selected, the algorithm assigns an action to each represented badge but with a flag marking them as uncertain.

*Compliance Algorithm*

The compliance algorithm calculates compliance from a set of opportunities and actions in three steps. The first step involves matching the actions to opportunities based on temporal proximity. In the second step, the algorithm filters out the opportunities matched to uncertain actions. Finally, the matched and unmatched opportunities feed into a calculation that determines the compliance.

*Matching Actions to Opportunities*

The matching algorithm uses the following criteria to determine which actions match to which opportunities: (i) an action can only match to a single opportunity; (ii) multiple actions may match to the same opportunity; (iii) a matching action and opportunity must occur within 90 seconds of each other; and (iv) an action cannot match an opportunity if there is an intervening opportunity. The algorithm determines each match in order from shortest to longest time between action and opportunity. When an action marked as certain (from the action algorithm) matches to an opportunity, the algorithm removes the opportunity from the potential match set so that no additional actions may match it. The end result is three types of opportunities: (i) no action matched; (ii) certain action matched; (iii) one or more uncertain actions matched. Figure 8 illustrates the matching algorithm.

*Filtering Out Uncertain Matches*

The subset of the opportunities matched to uncertain actions represents a case where the system did not have the discriminatory power to determine compliance behavior. To reduce error in the final compliance calculation, the compliance algorithm filters out these ambiguous data points. The remaining opportunities, those with certain actions or no action at all, are referred to as compliance data points.

*Compliance Calculation*

MedSense defines the compliance as the conditional probability of an action given an opportunity, denoted P(A|O). P(A|O) is equivalent to the joint probability of action and opportunity divided by the marginal probability of an opportunity, or P(AO)/P(O). Since the algorithm has removed the uncertain actions, this calculation becomes a weighted average where the action outcomes (zero or one) are weighted by the opportunity probabilities. This compliance calculation can be performed over any subset of the compliance data points, as is the case when calculating a compliance for a window of time, a category of HCWs, or any other grouping variable.

### The Pilot Trial

This study was conducted in the neurosurgical intensive care unit of Queen Mary Hospital in Hong Kong and was approved by the Institutional Review Board of the University of Hong Kong/Hospital Authority Hong Kong West Cluster on 8 October 2009. The trial consisted of two phases: an eight-month technical development phase (1 October 2009 to 30 May 2010) and a three-month evaluation phase (31 May 2010 to 31 August 2010). The technical development phase involved the initial deployment, calibration, and testing of the MedSense devices. Since the "WHO Guidelines for Hand Hygiene in Health Care" primarily address human observers, MedSense makes a number of assumptions that help span the gap between the capabilities of a human observer and an electronic, sensor-based system. The manufacturer worked with the hospital infection control team to set these parameters during the technical development phase. With the system fully installed and calibrated, the trial continued with the evaluation phase, which consisted of continuous system data collection and periodic unobtrusive hand hygiene observation by an infection control nurse.

**Figure 2:**
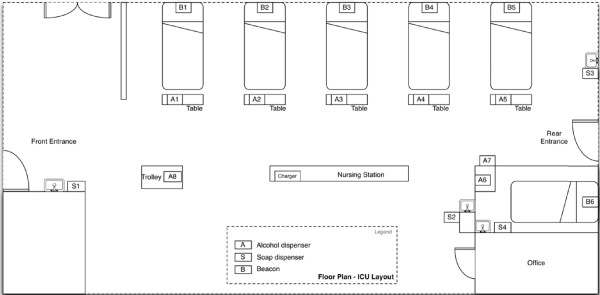
**Diagram of the trial unit showing the six patient beds with beacons behind them, eight alcohol dispensers instrumented with sensors, and four soap dispensers instrumented with sensors**.

The neurosurgical intensive care unit includes five patient beds in a common room and an additional bed in an adjoining side room. The common beds are spaced apart by approximately 1 to 1.5 meters along one side of the unit (Figure 2). The manufacturer mounted beacons on the wall at the head of each bed and configured them to create per-bed patient zones that covered the area within an arm's length of each bed. As HCWs can perform hand hygiene by either hand washing at one of four sinks or by hand-rubbing with alcohol-based handrub from dispensers located at the foot of each bed and around the unit, the manufacturer provided a pump bottle sensor for each of the soap and alcohol-based handrub dispensers. The sensor, which is designed as a holster for the product bottle, allowed the device to be either wall-mounted or placed on a flat surface.

The initial subject pool consisted entirely of HCWs who have direct contact with patients on the trial unit and included registered nurses, physicians, nursing officers, radiologists, physiotherapists, healthcare assistants, cleaners, and visitors. During the testing phase, about half of the target sample (i.e. physicians, radiologists, and visitors) had to be excluded due to their transient presence on the unit. The evaluation phase then proceeded with MedSense badges provided for only registered nurses, nursing officers, physiotherapists, and healthcare assistants.

Subjects who worked full-time on the unit received an individually named badge and included 13 registered nurses and two nursing officers. Subjects who worked part-time on the unit were provided with shared badges labeled by professional category, which included badges for four registered nurses ("Nurse 1" through "Nurse 4"), four physiotherapists ("Physio 1" through "Physio 4"), and a single healthcare assistant ("HCA"). The in-charge nurse was responsible for reminding the part time subjects to sign the badges in and out to prevent potential loss. In all, twenty-five badges were kept at the nursing station in two chargers for the evaluation phase.

We analyzed the overall compliance for the unit by averaging the daily compliance across all badges for each day of the evaluation phase. In addition, we determined per-indication compliance for Moment 1 and Moment 4, as well as compliance for opportunities that combined Moments 1 and 4. Opportunities considered for this two-indication group were mutually exclusive with the opportunities used in the single indication measures.

#### Comparing Human and System obtained Compliance Data

To evaluate the difference between compliance data captured by the system and by human observation, an infection control nurse trained to observe compliance with the WHO "My 5 Moments for hand hygiene" guidelines conducted a set of audit sessions on the unit while the system collected compliance data in parallel. We compared both the resulting activity index, defined as the number of opportunities per hour of patient care according to the previous publications [20-22], and compliance from each method. This analysis only included observed opportunities based on Moments 1 and 4 as these are the moments that the system can detect.

#### Comparing System Compliance with and without Observer on Unit

We tested whether the presence of an observer on the unit had an effect on the compliance as derived by the system. We compared the system's compliance during the observer audit sessions to the system's overall compliance for the days on which the audit sessions occurred.

#### System Compliance over Temporal and Subject Dimensions

Data obtained in the full evaluation phase were used to perform an exploratory analysis of the compliance over different dimensions. Temporal dimensions included time of day (by two hour time span), shift (AM from 06:30 to 13:45, PM from 13:45 to 20:45, and Night from 20:45 to 06:30), and day of the week. Subject dimensions included individual subject number and professional category (registered nurse, nursing officer, nurse using shared badge, physiotherapist using shared badge, and healthcare assistant using shared badge). In each of these cases, we calculated compliance for all the data within a group on a daily basis. For the case of professional category, the compliance were calculated per badge on a daily basis and then grouped into professional category.

#### Workload Analysis using Event Duration

Event duration (i.e. time between entry and exit from a patient zone) gives an indication of the unit workload because events measure the amount of time that the HCWs spend with patients. "Workload index", defined as the workload in terms of minutes spent within the patient zone per hour, was measured. We analyzed the cumulative workload index across all active subjects in the unit by time of day and by day of week to identify peaks and troughs.

### Statistical Analysis

For cases where data was limited, we used Fisher's Exact Test (FET) to determine if there were nonrandom differences between the compliance of two categories. In these cases, we treated the compliance data as binomial by considering the count of opportunities with and without actions. For analysis of the compliance and workload over the entirety of the evaluation data set, we used one-way analysis of variance (ANOVA) with a multiple comparison test (Tukey-Kramer) to determine differences in means where the ANOVA p-value was significant. For all tests, a p value of < 0.05 was considered statistically significant. Computation was performed using Matlab R2010b and the statistics toolbox.

## Results

### MedSense Setup

The study team successfully installed the MedSense system in the neurosurgical intensive care unit without causing electronic interference and without any disruption to ongoing work on the unit. The beacons were mounted on the wall behind the patient bed with industrial strength, double-sided Velcro. As battery powered devices, the beacons do not require access to wall power, which further simplified installation. Installing the tabletop pump bottle sensors took only moments, and the wall-mounted units required about a minute's worth of installation time as they have two screws that need to be screwed in place. The nursing station counter easily accommodated the two badge chargers, which each requires a connection to wall power. The team located a single base station at the rear of the unit, which covered the unit so that badges could upload their data within minutes of its being recorded.

### The Pilot Trial

The system collected compliance data for 93 days of the evaluation phase with an average of 4.99 (95%CI: 4.40, 5.58) badges reporting compliance data per day. All of the individually assigned badges and some of the shared badges collected compliance data. For the individually assigned badges, the 13 registered nurse badges each reported compliance data for an average of 23.7 days (95%CI: 16.31, 31.08), and the two nursing officer badges reported compliance data for 43 and 60 days. Of the shared badges, three physiotherapist badges reported compliance data for 8, 7, and 1 days; two shared nurse badges reported compliance data for 20 and 7 days; and the healthcare assistant badge reported compliance data for 10 days. The system detected 11,118 pairs of events when subjects entered and exited patient zones, which resulted in 16,327 hand hygiene opportunities. The compliance algorithm identified 13,694 of these opportunities as compliance data points, of which 4,702 had matching hand hygiene actions.

**Figure 3:**
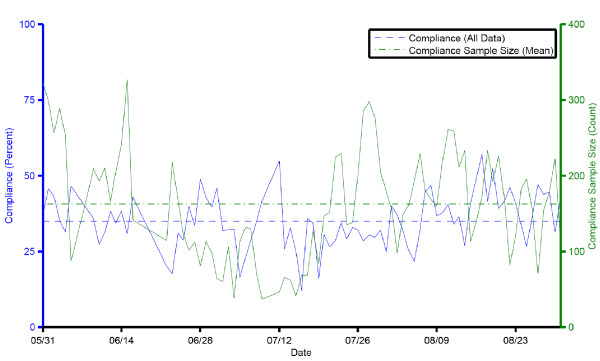
**Daily aggregate compliance and compliance data sample size shown over the course of the evaluation phase of the trial**.

We calculated the system compliance on a daily basis for the duration of the trial. The compliance for the unit sampled daily averaged 35.1% (95%CI: 33.2, 37.0). Figure 3 shows the daily aggregate compliance and compliance sample size over the course of the evaluation phase. The differences between the compliance for Moment 1 (21.3%, 95%CI: 19.0, 23.6), Moment 4 (39.6%, 95%CI: 37.3, 41.9), and joint Moments 1 and 4 (49.2%, 95%CI: 46.6, 51.8) were all statistically significant (p < 0.001).

#### Comparing Human and System Compliance Data

The observer conducted four 20-minute audit sessions of the unit over the course of one week (Table 2). Each was conducted between the hours of 10:00 and 11:15 am. Careful attention was paid to the start and end times of the observation sessions to ensure that the same time periods in the system collected data were compared. During the four audit sessions, the observer recorded a total of 69 opportunities (Moments 1 and 4) from 16 HCWs (four per session). Over this same period of time, the system recorded a total of 48 opportunities from 13 HCWs. Given that each session lasted 20 minutes, the observer recorded an activity index of 12.9 opportunities per hour, and the system detected an activity index of 11.1 opportunities per hour. Therefore, the system missed an average of 1.9 opportunities per hour, representing a percent error of 14.4%. The compliance determined by the system and observer were 88.9% (95%CI: 70.8, 97.7) and 95.6% (95%CI: 87.8, 99.1) respectively (FET, p = 0.34). The system miscalculated the observed compliance by 6.8%, which represents a percent error of 7.1%.

**Table 2:** Opportunities observed during direct observation sessions according to "My 5 Moments for Hand Hygiene"

Moment	First session	Second session	Third session	Fourth session	Total	Percent
Moment 1	8	9	10	13	40	49.3%
Moment 2	0	3	0	0	3	3.7%
Moment 3	2	1	3	2	8	9.8%
Moment 4	10	14	12	18	54	66.7%
Moment 5	0	0	4	4	8	9.8%
Opportunities	14	21	18	28	81	100%

The counts for the moments in the table are not mutually exclusive since an opportunity may represent more than one moment. For example, 69 of the 81 (85.2%) opportunities observed had at least one of Moments 1 and 4.

#### Comparing System Compliance with and without Observer on Unit

We compared the system's compliance during the audit periods to the overall system compliance on the days that the audits took place. Whereas the system detected 88.9% compliance during the four audit sessions, the overall compliance for the four days was 31.5%. This difference was statistically significant (FET, p < 0.001).

#### System Compliance over Temporal and Subject Dimensions

**Figure 4:**
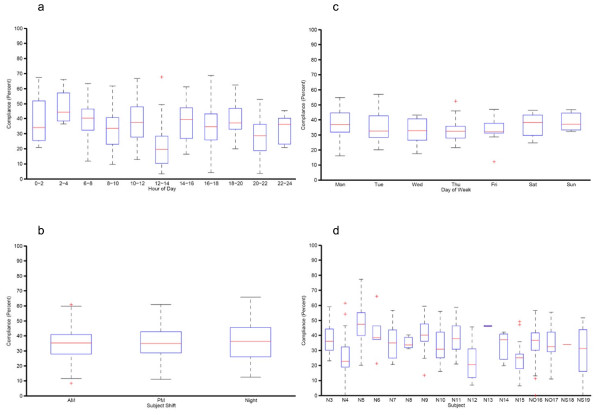
**Distribution of compliance according to different parameters**. **a**. Distribution of compliance calculated for two-hour periods of the day for each day of the evaluation phase and grouped by time period. **b**. Distribution of compliance calculated per shift on a daily basis and grouped by shift. Note. AM, morning shift from 06:30 to 13:45; PM, afternoon shift from 13:45 to 20:45; Night; night shift from 20:45 to 06:30. **c**. Distribution of compliance calculated on a daily basis and grouped by day of the week. **d**. Hand hygiene performance of individual subjects during the entire study period. Note. N, registered nurse; NO, nursing officer; NS, registered nurse with shared badge.

We analyzed an hourly presentation of the compliance data using time spans of two hours in duration. The hours from 12:00 to 14:00 (21.3%, 95%CI: 17.2, 25.3) had a notably lower compliance than other periods of the day (Figure 4a). The differences between this time span and 0:00 to 2:00 (38.9%, 95%CI: 30.1, 47.6), 2:00 to 4:00 (47.8%, 95%CI: 35.4, 60.1), 6:00 to 8:00 (39.5%, 95%CI: 35.4, 43.6), 8:00 to 10:00 (32.7%, 95%CI: 28.4, 37.0), 10:00 to 12:00 (37.6%, 95%CI: 34.3, 41.0), 14:00 to 16:00 (37.1%, 95%CI: 32.4, 41.7), 16:00 to 18:00 (35.0%, 95%CI: 31.2, 38.9), 18:00 to 20:00 (39.3%, 95%CI: 33.9, 44.7) were statistically significant (p < 0.001). Comparing across the AM, PM, and Night shifts, no significant effects were shown (Figure 4b). Compliance calculated daily and grouped by day of the week did not show any significant differences (Figure 4c).

**Figure 5:**
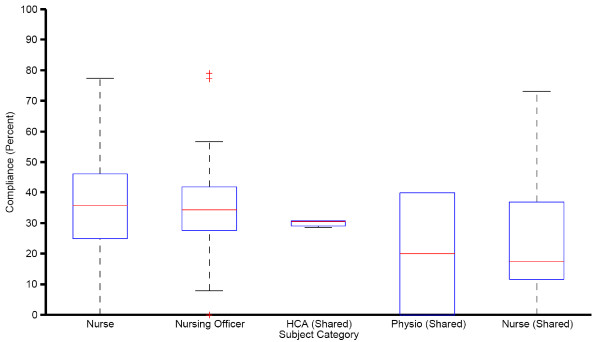
**Distribution of compliance calculated per badge and grouped by subject's HCW category**. Note. A single compliance is calculated per shared badge event though the subjects in these categories may change badges.

The hand hygiene performance of individual subjects is listed in Figure 4d. Grouping by professional category showed that the compliance of nurses who used shared badges (23.7%, 95%CI: 17.8, 29.6) was significantly lower (p < 0.01) than both registered nurses (36.1%, 95%CI: 34.2, 37.9) and nursing officers (34.0%, 95%CI: 31.1, 36.9) with assigned badges. Figure 5 shows the compliance calculated per subject's badge and grouped by category.

**Figure 6:**
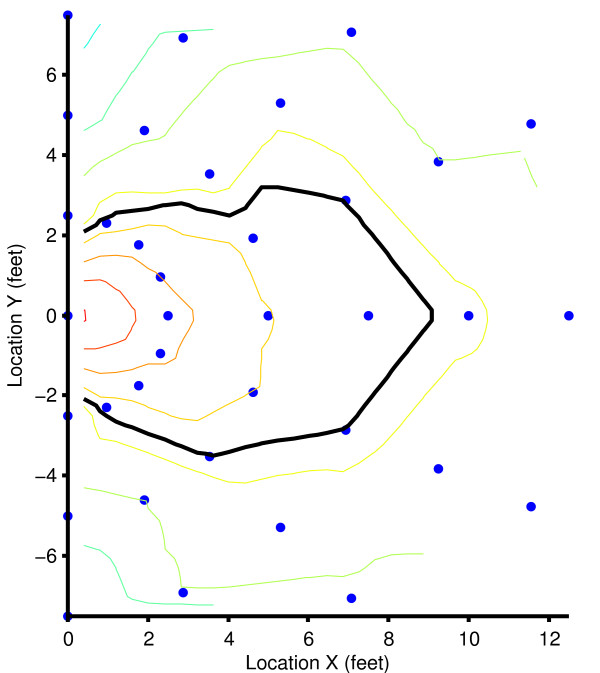
**The precision of the patient zone created by the beacon and plot of the beacon's transmitted field**. Note. The beacon is located at the head of the bed (x = 0, y = 0), and the bed extends out in the +x direction. The thin, colored lines represent contours of Received Signal Strength Indication (RSSI) measured by the badge and decreasing in signal strength at fixed intervals moving away from beacon's location. The thick, black line represents the threshold configured as the patient zone boundary.

**Figure 7:**
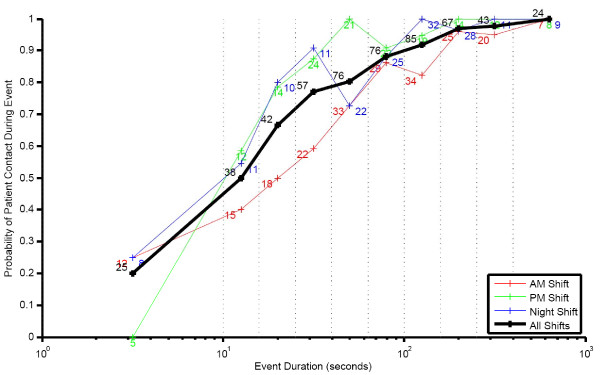
**Probability of patient contact during an event and event duration for three shifts on the unit**. Note. Number of samples is listed next to the data point

**Figure 8:**
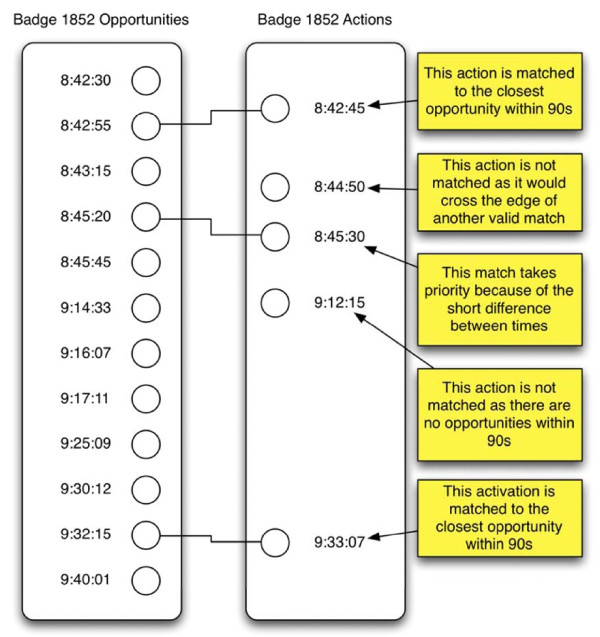
**The compliance algorithm matches actions to opportunities based on time**.

#### Workload Analysis using Event Duration

Analysis showed that workload index differed throughout the day. The hours from 14:00 to 16:00 (41.2, 95%CI: 37.0, 45.3) and 10:00 to 12:00 (39.0, 95%CI: 34.8, 43.3) had the highest workload index, and the hours of 4:00 to 6:00 (8.3, 95%CI: 2.5, 14.0) and 2:00 to 4:00 (10.6, 95%CI: 5.3, 15.9) had the lowest workload index. The differences between 32 combinations of two-hour bins were statistically significant (p < 0.001). The workload index did not differ in different days of the week.

## Discussion

We have successfully implemented an electronic monitoring system (MedSense) for compliance of hand hygiene based on the WHO "My 5 moments for hand hygiene" methodology in a neurosurgical intensive care unit with 6 beds of which 5 are in one cubicle. In contrast to the previously described electronic monitoring devices, which were mainly focused on recording the entries and exits of the HCWs and visitors to or from a single room or the measure of frequency of the alcohol or soap dispenser used; our current system can precisely calculate the hand hygiene opportunities in an open cubicle with multiple beds, which is strategically important in promoting infection control practice. The compliance of hand hygiene tends to be higher with patient care in single rooms with a door, which serves as a reminder to the attending HCWs to perform all necessary infection control measures [23]. Therefore, an electronic monitoring system targeted at patient care in open cubicles is urgently needed. The compliance measured by the system was closely correlated with the finding by manual observation. However, it is interesting to note that the system's compliance data showed that during the periods when the observer conducted the audit, the compliance was 2.8 times higher than the overall compliance for the same days. Similarly, hand hygiene compliance was significantly higher in the HCWs wearing designated badges than shared badges because their performance can be recognized. Intuitively, this makes sense as the HCWs may alter their behavior on knowing the presence of an observer [24]. Both of these observations may be explained by the Hawthorne effect which was also found in another study on the compliance with antiseptic handrub use in intensive care units [25]. Therefore, the overall hand hygiene compliance was only 35.1% for the study unit, which was lower than our previous reports of compliance to hand hygiene by direct observation [10,11,23]. Thus, the Hawthorne effect associated with the MedSense was less pronounced than that associated with the infection control nurses. The use of MedSense carries the advantages of reduced manpower requirement and provides an unobtrusive scientific tool for monitoring.

Whether the use of electronic monitoring and alert systems can make an improvement in hand hygiene practice remains controversial in the literature (Table 3). One prospective 1-month study conducted in a 30-bed hematopoietic stem cell transplantation & hematology unit demonstrated an improved compliance of hand hygiene from 36.3% to 70.1% after the provision of audible alert system to prompt HCWs to perform hand hygiene on 12 electronically monitored rooms upon entry and exit [14]. Another study conducted in a 14-bed intermediate care unit showed improved hand hygiene compliance by 37% when a voice-prompt system giving prerecorded messages reminded the individual to wash hands in one of the sinks and dispensers if they had not done so before exiting the room or within 10 seconds [12]. However, the use of electronic monitoring and feedback intervention did not reveal any significant change in hand hygiene compliance in two 20-bed step-down units [15]. In fact, the most recent study on the electronic monitoring device being installed in 20 patient rooms' entrance and 70 dispensers for soap or hand sanitizer in 5 patient care units of a territory hospital in US reported the hand hygiene compliance was as low as 25% without any feedback intervention [18]. Therefore, further investigation has to be performed to understand if a sustained improvement in hand hygiene practice can be achieved by the use electronic monitoring system.

**Table 3:** Review of literature on the use of electronic device in monitoring hand hygiene

Study [reference]	Design, setting, and main intervention	Major outcome and remark
Swoboda SM et al (2004) [12]	Prospective 14-month study in a 14-bed intermediate care unit, Baltimore, US; Electronic monitoring system to record entry and exit from patient rooms, the use of toilet and dirty utility facilities, and the use of hand washing and hand hygiene devices; Phase 1 (6-month): electronic monitoring and 8-weeks of direct observation of staff interactions; Phase 2 (6-month): voice-prompt system giving prerecorded messages to remind the individual to wash hands if they had not done so before exiting the room or within 10 seconds in one of the sinks and dispensers; Phase 3 (2-month): electronic monitoring without voice-prompt system	Hand hygiene compliance in patient rooms improved by 37% during phase 2 and 41% in phase 3; while the number of infection decreased by 22% and 48% in the corresponding period; Patient care practice was not precisely observed
Kinsella G et al (2007) [13]	A 47-day study in a 16-bed ICU, Salford, UK; Electronically record the use of wall-mounted soap and alcohol gel dispensers implanted in two bed areas and entrance of ICU; Measure the consumption pattern of wall-mounted soap and alcohol gel dispenser	Consumption of alcohol gel dispenser in bed area was correlated with the dependency of the patient (r = 0.5, p < 0.01); Compliance of hand hygiene was not measured
Venkatesh AK et al (2008) [14]	Prospective 1-month study in a 30-bed hematopoietic stem cell transplantation & hematology unit, Chicago, US; Audible alert to prompt healthcare workers to perform hand hygiene on 12 electronically monitored rooms upon entry and exit; Phase 1 (2-week): monitor baseline compliance of hand hygiene Phase 2 (2-week): monitor hand hygiene compliance with automatic alerts	Improved compliance of hand hygiene from baseline (36.3%) to 70.1% during phase 2; Patient care practice was not precisely observed
Marra AR et al (2008) [15]	A 6-month control trial in two 20-bed step-down units, Sao Paulo, Brazil; Electronic counting devices for wall-mounted alcohol gel dispensers were available in two step-down units, one with feedback intervention program and one without (control)	No significant difference in the amount of alcohol gel used and hand hygiene compliance; Patient care practice was not precisely observed
Boscart VM et al (2008) [16]	Descriptive study in teaching facilities, Ontario, Canada; The wearable electronic monitoring device communicated with the alcohol gel dispensers and patient zone to provide signal to perform hand cleansing; The acceptability and usability of wearable electronic hand wash device was assessed	All ten staff accept the use of the electronic device; An individual patient environmental zone was defined
Boyce JM et al (2009) [17]	Prospective observation trial for 6-month in a 22-bed general medical ward and a 15-bed surgical ICU, New Haven, US; Electronic device was used to record the frequency of dispenser used	The dispenser located in patient rooms account for 47% and 36% of hand hygiene events performed in surgical ICU and general medical ward respectively; The hand hygiene event was indirectly measured by the dispenser used. The compliance of hand hygiene was not assessed
Sahud AG et al (2010) [18]	A 2-phase pilot study in 5 patient care units of a territory hospital, Pittsburgh, US; Electronic device was installed in 20 patient room entrances and 70 dispensers for soap or hand sanitizer; Phase 1 (8-month): manual observation at patient room entry and exit Phase 2 (4-week): observation using electronic device	Electronic device captured 98% of manually recorded room entries and 95% of dispensing event; The compliance was low (25.5%)
Edmond MB et al (2010) [26]	A 2-phase study in a 35-bed orthopedic ward, Virginia, US; Volunteered nursing staff wore a credit-card-size alcohol sensor badge, which can detect alcohol vapor upon room entry or exit; if alcohol vapor was not detected within 8 s, the badge light would turn red and produce "beep" sound Phase 1 (21 days): direct observation of hand hygiene compliance Phase 2 (10 days): observation using electronic device	Compliance of hand hygiene among nursing staff increased from 73% in phase 1 to 93% in phase 2 (p = 0.01) The system only measured compliance on room entry and exit; the hand hygiene opportunities occurred inside patient room were missed
Polgreen PM et al (2010) [27]	Description of an electronic device of small credit-card-sized without radio-frequency identification to monitor the use of hand hygiene dispensers before healthcare workers enter or exit patient rooms	No clinical data being mentioned

ICU, intensive care unit

Since the electronic monitoring system is non-intrusive for HCWs and allows information to be continuously collected throughout the day, it can provide concrete data on the performance of individual staff or clinical units at any given time. For instance, the compliance of each HCW can be assessed and compared with the aggregated compliance of all the team members. Positive reinforcement with award can be accorded to HCW who has sustained achievement in hand hygiene, whereas further reinforcement by infection control team can be provided for those who have not complied with the hand hygiene recommendations. As the confidentiality of data is ensured, the system is acceptable to our HCWs, which is similar to the report from Boscart VM et al [16]. As another example, we have identified an interesting intraday pattern that the hand hygiene compliance was significantly lowered to 21.3% between the hours of 12:00 and 14:00. This might be attributable to the rush to finish off work within the period as the morning shift ended at 13:45. The system could report this information to the infection control team so that the issue can be further investigated.

There are several limitations in this study. Firstly, the electronic sensor was not installed in the pocket size alcohol-based handrub during the initial phase of this study. Hand hygiene action when using pocket size alcohol-based handrub has not been analyzed, which may underestimate the overall hand hygiene compliance among our staff. However, we advised our staff to use the bottle size alcohol-based handrub inside the area during the study period. Secondly, the compliance algorithm was configured to only match hand hygiene actions to opportunities when the action occurred at most 90 seconds before or after the opportunity. This may in some cases lead to positive opportunities being incorrectly classified, which would negatively bias the system's compliance. However, the 90-second matching span is not a technical limitation of the system but rather a configurable parameter that was selected based on intuition and the results of a sensitivity analysis of manual observations. Thirdly, while the system can detect the HCW's entry and exit to and from patient zones, it cannot definitively determine the actual sequence of hand hygiene and patient contact, especially when hand hygiene occurs during the event. This is a limitation to the method of inferring patient contact over directly observing it. However, all methods of observation are subject to uncertainty, and in most circumstances, the manual observation closely correlates with the system's findings in the correct sequence. Fourthly, doubt might exist on whether the predefined reference table (described in Table 1) used to predict the probability of patient contact having occurred during an event, which was built based on observations, might be different from the "normal setting". However, in reality, the two were actually quite similar. In order to establish the predefined reference table, observations on the duration of direct patient contact time during various patient care clinical activities were made. The staff performed routine patient care activities during the observational period which were similar to the "normal setting". Finally, we only performed four sessions of hand hygiene observation during the evaluation phase, which may not be able to fully demonstrate the discriminatory power of the electronic system. A three-month evaluation phase is a rather short period. We only obtained baseline data during the study period without making any intervention and may not be able to show any change in incidence of nosocomial infection as reported in the previous study [12]. Though at this stage of its development the cost of this system is not yet defined, this issue remains a concern for resource limited settings. Therefore, a cost-effectiveness analysis must be conducted before the system is to be installed in all clinical units of the hospital. However, priority should be given to those high-risk areas such as intensive care units, coronary care units, and blood and marrow transplantation centers.

## Conclusions

Since MedSense can continuously monitor the activity of different professional categories without disturbing their patient care practice, the performance of the individual staff or clinical units can be reviewed from time to time by their respective supervisor, infection control team and the hospital administration. Therefore, timely feedback and intervention can be introduced to enhance the compliance of hand hygiene practice.

## Competing interests

The authors declare that they have no competing interests.

## Authors' contributions

VCCC, JWMT and KYY designed, executed and supervised the study. SKYH performed hand hygiene observation. VCCC drafted the manuscript. JFWC, KNH, and PLH critically reviewed the manuscript. All authors have read and approved the final manuscript.

## Pre-publication history

The pre-publication history for this paper can be accessed here:

http://www.biomedcentral.com/1471-2334/11/151/prepub
